# Description and molecular data for a new acanthocephalan parasite, *Polymorphus circi* n. sp. (Polymorphidae) from the Australasian harrier (*Circus approximans* Peale) in New Zealand

**DOI:** 10.1007/s11230-023-10120-5

**Published:** 2023-10-24

**Authors:** Bronwen Presswell, Jerusha Bennett

**Affiliations:** Department of Zoology, University of Otago, PO Box 56, Dunedin, New Zealand

## Abstract

Species of genus *Polymorphus* Lühe, 1911 (Polymorphidae) are acanthocephalans found in fish-eating birds and waterfowl. Although found in many parts of the world, including Australia, no records exist from New Zealand. Because of the largely aquatic intermediate host, *Polymorphus* species are rarely found in terrestrial birds of prey. During a study of the helminths of the Australasian harrier *Circus approximans* Peale specimens of *Polymorphus* were recovered that were found to be new to science. *Polymorphus circi* **n. sp.** is formally described and genetic sequence data presented. Specimens were distinguished from all other species by a combination of characters, including their proboscis hook arrangement (20–22 rows of 11–13 hooks), as well as absence of sexual dimorphism, trunk size, proboscis shape and egg size. These acanthocephalans were found in birds from areas with the potential to support freshwater, brackish or marine amphipods, but as yet the actual intermediate hosts are unknown.

## Introduction

The genus *Polymorphus* Lühe, 1911 (Acanthocephala: Polymorphida: Polymorphidae) is a widespread group of parasite species that infect the gastrointestinal tract of fish-eating birds and waterfowl, and use amphipods as intermediate hosts (Crompton & Nickol 1985; Amin 1992). Species of *Polymorphus* have been recorded from Europe, North and South America, Russia and Pakistan, and one of the 36 known species is found in Australia (Amin 1992; Johnston & Edmonds 1948). Apart from an unnamed larval stage in a hedgehog (Smales et al. 2010), there are no records from New Zealand. The taxonomic history of genus *Polymorphus* is complicated, with various other genera having been treated as synonyms (*Profilicollis* Meyer, 1931*, Falsifilicollis* Webster, 1948, *Parafilicollis* Petrochenko, 1956, *Subfilicollis* Hoklova, 1967, *Subcorynosoma* Hoklova, 1967*, Arhythmorhynchus* Lühe, 1911*, Hexaglandula* Petrochenko, 1950) or as subgenera (*Profilicollis, Hexaglandula*) in earlier taxonomic treatments (Yamaguti 1963; Amin 1992). The advent of phylogenetics has enabled some clarity, confirming the generic identity of *Profillicollis, Arhythmorhynchus* and *Hexaglandula* (García-Varela et al. 2011, 2013; García-Varela & Pérez-Ponce de León, 2008). However, all of these studies produce phylogenetic hypotheses in which available sequences of *Polymorphus* are polyphyletic, indicating that species need to be re-examined and reclassified using morphological and ecological data, and a broader molecular range.

The Australasian harrier *Circus approximans* Peale (Accipitriformes: Accipitridae), also known as swamp harrier, harrier hawk or kāhu, is native to Australia, New Zealand and some islands in the South Pacific (Debus & Kirwan 2020). It is an opportunistic hunter of live prey such as small birds, mammals and invertebrates, and also a scavenger, with carrion making up a major component of the diet (Baker-Gabb 1981). In New Zealand, harriers hunt mainly in open habitats, and the population has benefitted from widespread deforestation for agriculture. Along with the ready availability of road-kill carcasses, this has seen the harrier rise to healthy population numbers (Eakle 2008). Its conservation status is Not Threatened (Robertson et al. 2021), but the bird is considered a taonga (“treasured”) species by Māori, and is partially protected by law (Wildlife (Australasian Harrier) Notice 2012). Many harriers are themselves victims of roadkill or injury (Sadleir & Linklater 2016), meaning considerable numbers are available for study. In Australasian harriers, only one acanthocephalan has been reported in Australia (*Centrorhynchus asturinus* (Johnston, 1913) in Smales 2003), and no records exist for the New Zealand population.

The opportunity to examine a large number of deceased harriers from the southern half of South Island since 2017 has allowed the authors to conduct a survey of all helminth parasites found, and what follows is a description of a polymorphid acanthocephalan found in some birds, which was found to be new to science. We provide 28S rDNA sequence for future comparison. A description of a new species of cestode and a report on other helminths recovered from the NZ harriers, including a new species of nematode, will be published elsewhere.

## Materials and methods

### Harrier collection and processing

A total of 65 harriers was examined: 41 from Otago, donated by the Dunedin Wildlife Hospital between 2017 and 2022; 19 from Canterbury, donated by The New Zealand Raptor Trust in 2022/3, and five collected as roadkill by the first author. Birds were frozen for storage, defrosted prior to dissection, and helminths were collected and preserved in 70% ethanol for whole-mount, 96% ethanol for genetic analyses and 4% buffered formalin for SEM imaging.

### Morphological data

Acanthocephalan specimens were cleared and mounted temporarily in beechwood creosote for photography. Measurements were made using ImageJ software (Wayne Rasband, NIH, USA) from photographs taken on an Olympus BX51 compound microscope mounted with DP25 camera attachment (Olympus, Tokyo). All measurements are in micrometres unless otherwise indicated, and in descriptions are given as range, followed by mean in parentheses, where numbers permit. To compare overall size between sexes a two-tailed Student’s t-test was computed in Excel (Microsoft, 2017). Drawings were made from photographic series.

Specimens chosen for scanning electron microscopy (SEM) were transferred to 2.5 % gluteraldehyde in 0.1 M phosphate buffer, post-fixed in 1% osmium tetroxide and dehydrated through a gradient series of ethanols, critical-point dried in a CPD030 BalTec critical-point dryer (BalTec AG, Balzers, Liechtenstein) using carbon dioxide, mounted on aluminium stubs, and sputter coated with gold/palladium (60:40) to a thickness of 10 nm in an Emitech K575X Peltier-cooled high-resolution sputter coater (EM Technologies, Ashford, Kent, UK). The specimens were viewed with a JEOL 6700 F field emission scanning electron microscope (JEOL Ltd., Tokyo, Japan) at the Otago Centre for Electron Microscopy (OCEM, University of Otago, New Zealand).

### Molecular data and analyses

Genomic DNA from one individual was extracted using the DNeasy® Blood & Tissue Kit (Qiagen, Hilden, Germany) according to the manufacturer’s protocol. A partial fragment of 28S rRNA gene was amplified using T16 and T30 primers (Harper & Saunders, 2001) and conditions following Bennett et al. (2023). PCR products were cleaned using EXOSAP Express PCR Product Cleanup Reagent (USB Corporation, Cleveland, OH, USA), following manufacturer’s instructions. Sanger sequencing by capillary electrophoresis was performed by the Genetic Analysis Service, Department of Anatomy, University of Otago (Dunedin, New Zealand).

The successfully amplified sequence was imported into Geneious Prime®v1.2, trimmed using the trim function with default parameters, and manually edited for incorrect or ambiguous base calls. An alignment was created with the new sequence and other closely related species and representatives from genus *Polymorphus*. Uncorrected pairwise genetic divergences within genus and between species were calculated in MEGA v.11 and the resulting 28S sequence was deposited with GenBank under accession OR593504.

## Results

A total of 50 acanthocephalans was found in 4 birds. Using the key to the genera of the Polymorphidae of Presswell et al. (2020), the specimens clearly key out to genus *Polymorphus*. A consideration of the numbers of hook rows and number of hooks per row, size of adult worms, presence or absence of sexual dimorphism, egg size, genetic sequences and geographical locality found no nominal species comparable to the New Zealand specimens, and they were adjudged to represent a species new to science, which is described below.

**ACANTHOCEPHALA Koehlreuther, 1771**

**Polymorphida Petrochenko, 1956**

**Polymorphidae Meyer, 1931**

***Polymorphus Lühe, 1911***

**Fig. 1:**
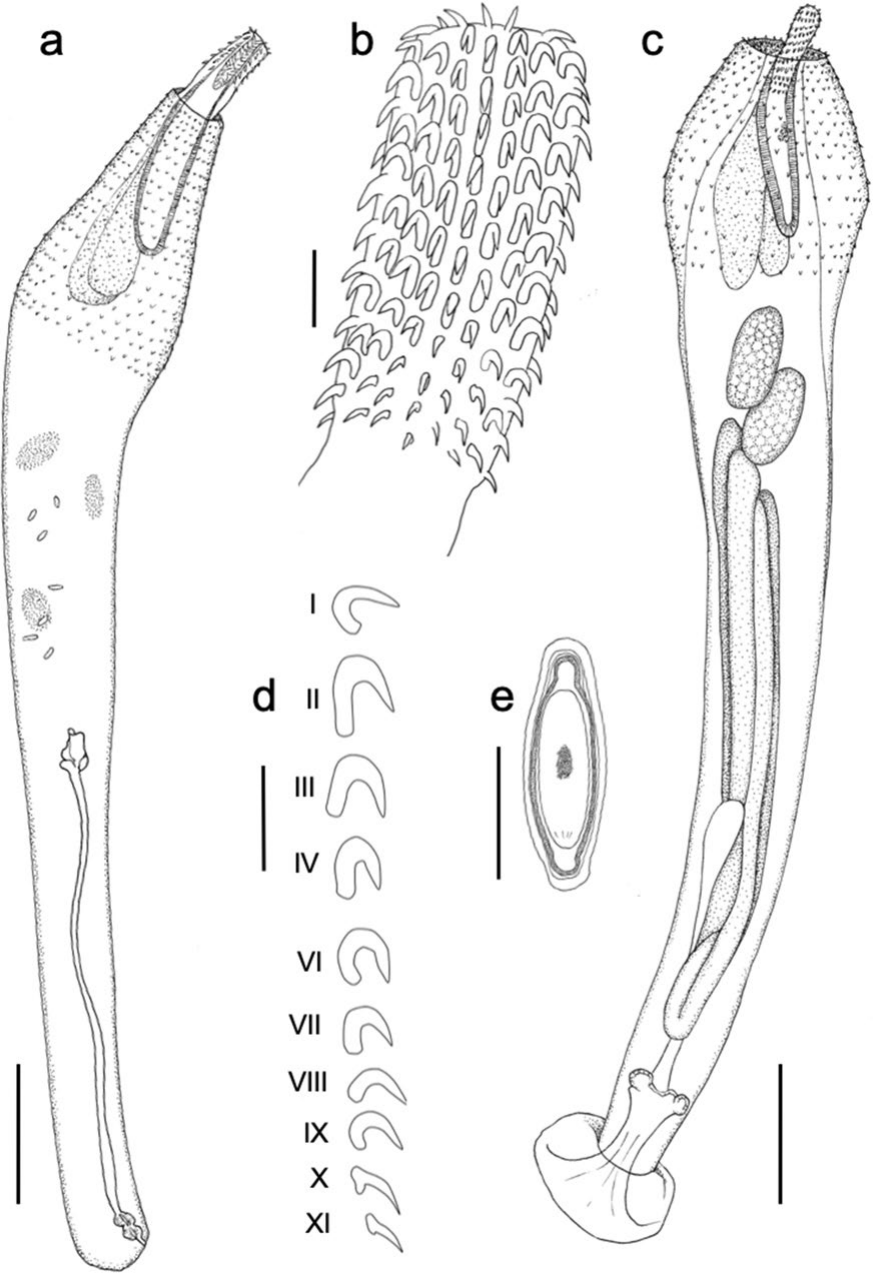
*Polymorphus circi* **n. sp.** (a) Whole female with partly inverted proboscis, (b) proboscis, (c) Whole male, (d) Proboscis hook row. Note that hook number V is missing, (e) Egg. Scale bars: a and c = 1 mm; b = 100 µm; d and e= 50 µm.

**Fig. 2:**
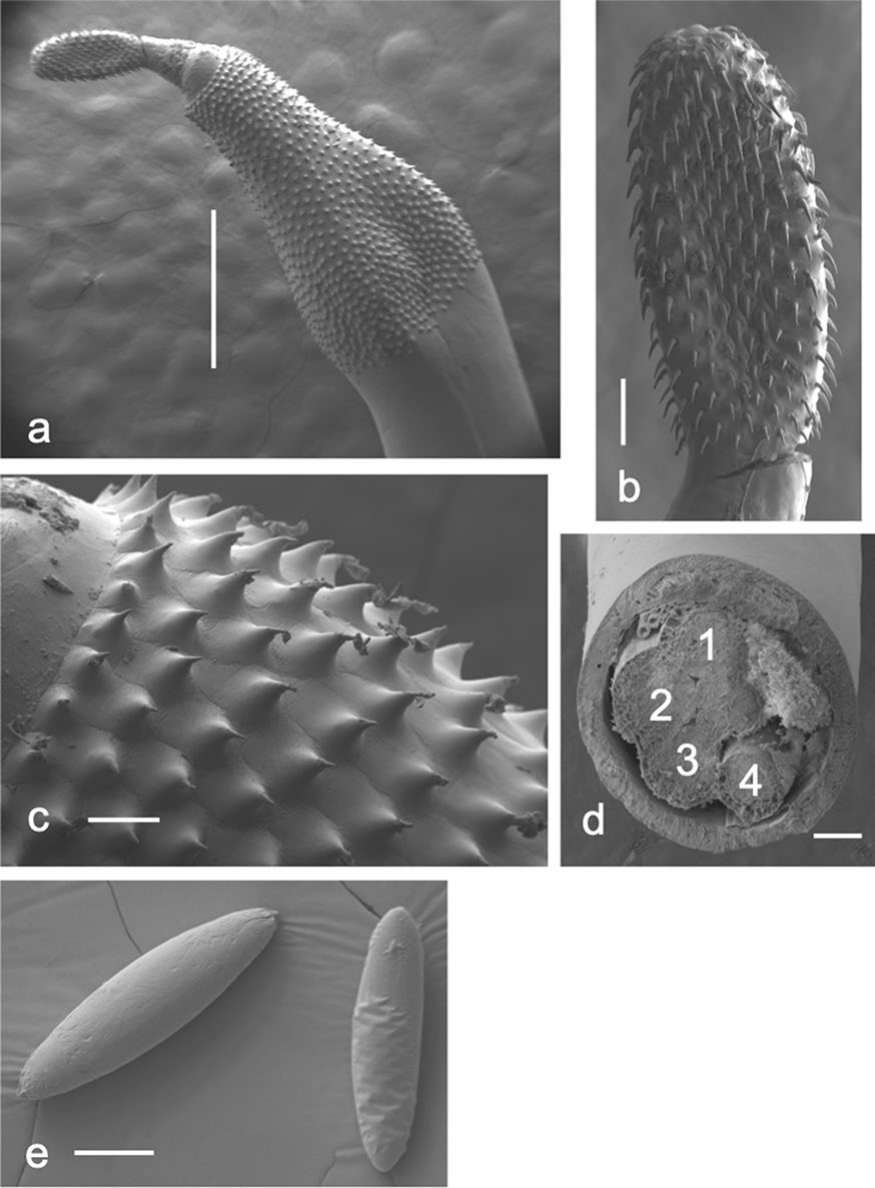
*Polymorphus circi* **n. sp.** scanning electron micrographs. (a) praesoma showing distribution of trunk spines, (b) proboscis, (c) higher magnification of trunk spines, (d) cross-section of male through cement glands (numbered 1 to 4), (e) Eggs. Scale bars: a = 1 mm; b and d= 100 µm; c = 50 µm; e = 20 µm.

**Fig. 3:**
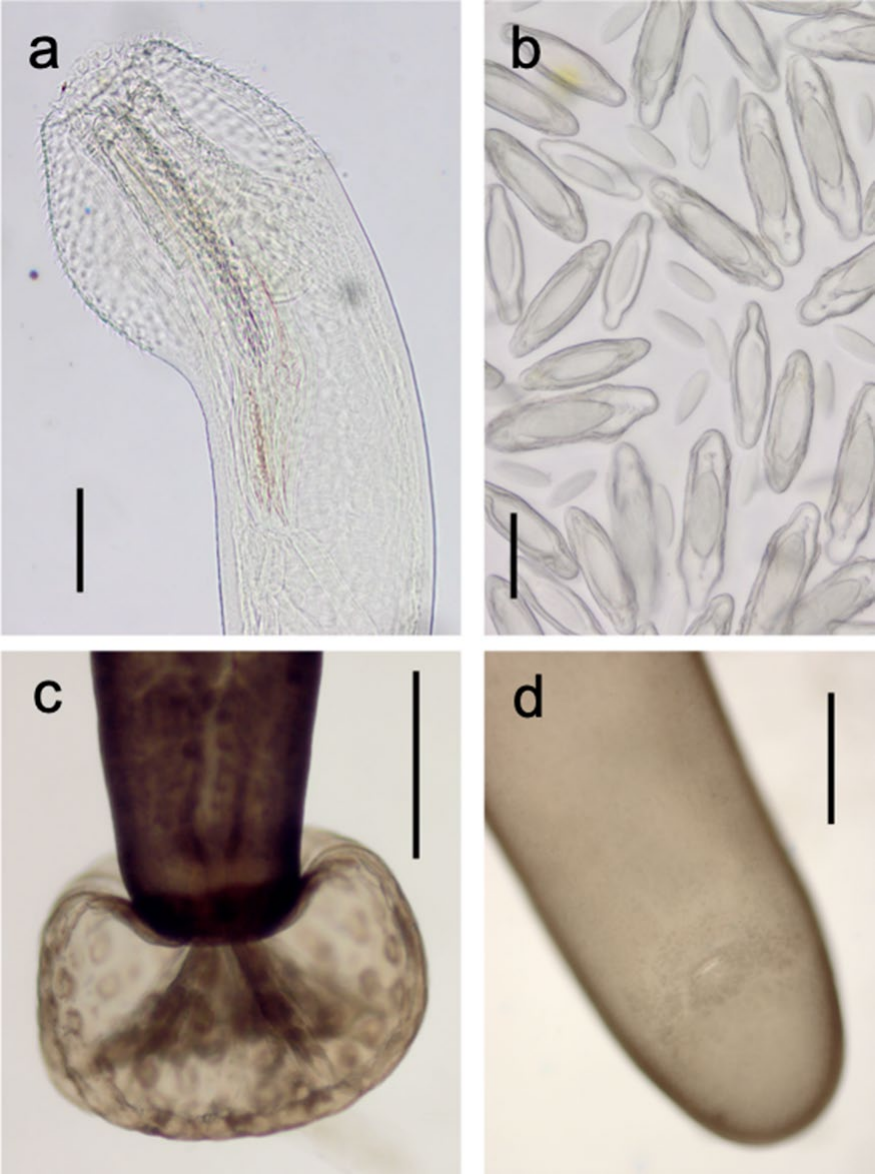
*Polymorphus circi* **n. sp.** photomicrographs. (a) Cleared immature specimen with inverted proboscis, (b) eggs in all stages of development, (c) partly everted copulatory bursa, (d) female genital pore showing non-terminal position. Scale bars: a = 200 µm; b = 50 µm; c and d = 500 µm.

***Polymorphus circi n. sp. (***Figures 1, 2 and 3**)**

*General* [based on 25 specimens]: With characters of the genus. Cream in colour when removed from intestine. No significant sexual size dimorphism (two–tailed t–test, t(17) = -1.128, P>.05 based on mean length of adult worms). Trunk cylindrical, with anterior expansion for approximately one quarter of worm length in both sexes. Anterior expansion with single field of spines in 23–25 circles; spinous field equally long dorsally and ventrally; spines more or less uniform in size, 44–55 long, and evenly spaced in a fully extended specimen. Hypodermal nuclei extend from about middle of trunk spine field to near the posterior end of trunk. Neck conical, broadest at base, about two thirds as long as proboscis in fully extended specimens. Proboscis cylindrical, with 20–22 rows of 11–13 hooks; hooks more or less equal in size throughout, but posteriormost two or three finer. Roots simple, longer than blades in anterior hooks, small with anteriorly directed manubria in posteriormost 2 or 3 hooks. Proboscis receptacle inserted at base of proboscis, double walled, with cerebral ganglion at mid–point. Lemnisci clavate, longer than proboscis receptacle.

*Adult male* [based on 11 specimens]: Trunk 8.20–9.93 (9.21) mm long by 0.97–1.59 (1.20) mm at widest part of expansion. Spine field 1.20–1.96 mm long. Proboscis 544–676 (611) long by 196–235 (210) wide at middle; 20–22 longitudinal rows of 11–13 hooks each; hook lengths [root lengths] in 1 male measured from proboscis anterior, 36 [–], 38 [39], 40 [43], 40 [43], 36 [45], 41 [43], 42 [51], 38 [–], 39 [–], 40 [17], 27 [12]. Neck 240–401 (342) long. Proboscis receptacle 949–1,184 (1090) long by 199–304 (251) wide. Lemnisci 1,089–1,579 (1,345) long by 214–356 (299) wide posteriorly. Testes ovoid, oblique, overlapping, close to posterior end of lemnisci, sometimes overlapping lemnisci; anterior testis 505–864 (744) long by 316–506 (419) wide, posterior testis 510–1,016 (741) long by 339–513 (442) wide. Cement glands 4 (Fig. 2d), tubular, unequal lengths, reaching as far as posterior testis; longest 3,454–6,145 (4,875) long by 180–247 (210) wide. Saefftigen’s pouch 922–1244 (1022) long. Posterior extremity rounded, with terminal genital pore. Copulatory bursa with many sensory pits, length 667–865, width 749–1194 (n=3) (Fig. 3c), size dependent upon stage of eversion.

*Adult female* [based on 8 specimens]: Trunk 7.78–9.99 (8.89) mm long by 1.13–1.48 (1.30) mm at widest part of expansion. Spine field 1.42–1.86 mm long. Proboscis 556–821 (670) long by 210–232 (221) wide at middle, 20–22 longitudinal rows of 10–13 hooks each; hook lengths [root lengths] in 1 female measured from proboscis anterior, 31 [36], 42 [45], 42 [44], 45 [50], 41 [49], 38 [45], 40 [39], 36 [36], 35 [21], 30 [20], 31 [–]. Neck 363–403 (383) long. Proboscis receptacle 911–1,267 (1,045) long by 211–298 (264) wide. Lemnisci 1,336–1,801 (1,574) long by 308–324 (316) wide posteriorly. Posterior extremity rounded, sometimes slightly inflated on ventral aspect; genital pore sub-terminal, on ventral surface (Fig. 3d). Female reproductive tract difficult to observe, obscured by eggs in older specimens. In one female, of 8.2 mm long, entire reproductive system 3.5 mm long, vaginal complex 690 long. Eggs elongate with polar prolongation of middle membrane, outer shell 80–111 (89) long by 21–27 (23) wide.

*Immature male* [based on 2 specimens]: Specimen with reproductive organs present, but in early development, proboscis and bursa inverted. Trunk 2.35–2.36 mm long, 526–535 wide. Proboscis (inverted) 409 long (n=1); proboscis receptacle 525–716 long, 159–189 wide. Testes small and almost parallel. Anterior testis 322 long, 125 wide; posterior testis 307 long, 148 wide (n=1).

*Immature female* [based on 1 specimen]: Specimen with no visible signs of reproductive tract, ovarian balls or eggs. Trunk 2.57 mm long, 546 wide. Proboscis (part inverted) 486 long, 192 wide; proboscis receptacle 812 long, 189 wide. Lemnisci folded and crumpled to appear shorter than proboscis receptacle.

*Type host.* Australasian harrier *Circus approximans* Peale (Accipitriformes: Accipitridae).

*Type locality.* Timaru, Canterbury District, South Island, New Zealand. 44°23′54″S, 171°15′36″E.

*Other localities.* Maungatua and Waihola, Taeiri plain, Dunedin, South Island, NZ. 45°55', 170°09'.

*Site of infection.* Intestine*.*

*Prevalence and intensity.* In four birds out of 65 (6%), intensity 1 to 40 (mean 12.5)

*Type material.* Holotype male W.003957 Allotype female W.003958 Paratypes, 5 specimens W.003959 from Taieri Plain: deposited with the Museum of New Zealand, Te Papa Tongarewa, Wellington, NZ.

*Voucher material*. Hologenophore W.003960

*Representative DNA sequence*. Genbank accession OR593504

*Zoobank taxon reference.* 43CC3935-AECB-47D9-B06C-D94C5BE57E6E

*Etymology*. The specific name “*circi”* (a masculine noun in genitive case) relates to the genus of the unusual definitive host.

### Remarks

This polymorphid acanthocephalan exhibits a number of characters that define it as a species of *Polymorphus*: trunk cylindrical with anterior expansion, foretrunk with a single field of spines, proboscis cylindrical to ovoid, hooks slightly larger in middle, proboscis receptacle double-walled and inserted at base of proboscis, ganglion near middle of receptacle, lemnisci clavate, testes in anterior half of trunk, four tubular cement glands, eggs with polar prolongation of middle membrane (Amin 1992; Presswell et al. 2020).

Despite considerable historical disagreement over the members of this genus, 36 species of *Polymorphus* are currently recognised in the literature (Amin 2013). Of these nearly all are reported from ducks, waders and other freshwater and marine birds. Four species are recorded from accipitriform birds: *P. brevis* (van Cleave, 1916) Travassos 1926, *P. magnus* Skrjabin, 1913, *P. meyeri* Lundström, 1942 and *P. striatus* (Goeze, 1782) Lühe 1911. Each of these records is however supplemental to more frequent reports of aquatic bird hosts, and three of the records involve *Haliaeetus* spp. which are fish-eagles. Only one record involves a truly terrestrial raptor host; *P. magnus*, which was found in the tawny eagle *Aquila rapax* (Temminck), an accipitrid with a scavenging habit similar to *Circus approximans* (Ferguson-Lees & Christie 2020)*.* Of those species found elsewhere in raptors, none shares the same hook arrangement as *P. circi* **n. sp.** (20–22 rows of 11–13 hooks): *P. brevis* has 18 rows of 15 hooks*, P. magnus* has 14–18 rows of 7–9 hooks*, P. meyeri* has 16–17 rows of 6 hooks*,* and *P. striatus* has 16 rows of 12–16 hooks.

Only one species has been recorded from the nearest landmass, Australia: *P. biziurae* Johnston & Edmonds, 1948, found in a musk duck *Biziura lobata* (Shaw); it has a similar hook arrangement (21–22 rows of 9–11 hooks) to *P. circi* **n. sp.**, but differs in its sexual size dimorphism, greater trunk length of the female (1.7 mm–18.2 mm), greater length of the proboscis receptacle (1.2–2.3 mm) and smaller size of the eggs, which lack polar prolongations.

All of the other nominal species differ from *P. circi* **n. sp.** in their proboscis hook arrangement, except for *P. cucullatus* van Cleave & Starrett, 1940, *P. mathevossianae* Petrochenko, 1949, *P. spindlatus* Amin & Heckmann, 1991, and *P. swartzi* Schmidt, 1965. *Polymorphus cucullatus* (22 rows of 12 hooks) is a larger worm (males 11-13 mm, females 10 mm) with a pyriform proboscis rather than cylindrical, and much larger proboscis hooks (maximum length 85 µm as opposed to 51 µm in* P. circi* **n. sp.**, *P. mathevossianae* (20 rows of 11–12 hooks) is much smaller in size (males and females 2.6–4.5mm) and has a very long field of trunk spines (60 circles), *P. spindlatus* (18–20 rows of 11–13 hooks) has a distinctive spindle-shaped proboscis and is much smaller in size (males 3.5–5.2 mm, females 3.6–6.0 mm), and *P. swartzi* has a swollen proboscis and is also smaller (female only described, 4.7 mm).

### Genetic results

Our newly generated 28S sequence of *Polymorphus circi* **n. sp.** was closest to *Polymorphus trochus* from an American coot *Fulica americana* GenBank accession JX442185 (García-Varela et al. 2013) with 37.1% genetic divergence at 28S. The average genetic divergence observed between species within *Polymorphus* was 41.0%, ranging between 34.2-51.5%. This comparison included available representatives from GenBank, including *Polymorphus brevis* AY829105 and JX442183, *P. trochus* JX442185, *P. minutus* EU267819, *P. obtusus* JX442184 and *Polymorphus* sp. AY829109. These large divergences illustrate the considerable variability in the 28S gene for these acanthocephalans. For instance, García-Varela et al. (2013) used Pro-Align to detect and remove unreliably aligned sections from their polymorphid 28S alignment, which resulted in a 26% loss of sites. Because the sequences were essentially unalignable, any phylogenetic inferences were unreliable, so a tree is not presented here. Amplification of three other genes (18S, ITS and *cox1*) was unfortunately unsuccessful.

## Discussion

No named species of *Polymorphus* has been recorded in New Zealand previously, but there are two reports of unidentified species. McDonald (1998) noted the presence of *Polymorphus* sp. in a pied stilt *Himantopus leucocephalus* Gould. However, McDonald’s specimens were initially identified as belonging to genus *Falsifilicollis*, which, following a tortuous history of synonymisation and subsumption, is now regarded as *Profilicollis* (García-Varela & Pérez-Ponce de León 2008). Smales et al. (2010) also recognised the stilt record as *Profilicollis* sp. A second report of *Polymorphus* sp. in New Zealand was of an acanthella (pre-encysted juvenile stage) from a hedgehog *Erinaceus europaeus* L*.* (Smales et al. 2010). All paratenic hosts of acanthocephalans are vertebrates (Kennedy 2006), and hedgehogs have been recorded as paratenic hosts for a related acanthocephalan, *Plagiorhynchus cylindraceus* (Goeze, 1782), both in New Zealand and in their native Europe (Skuballa et al. 2010). *Plagiorhynchus cylindraceus* has a non-aquatic life cycle that includes terrestrial isopods as intermediate hosts and passerine or corvine birds as definitive hosts (Skuballa et al. 2010), so infection of a bird scavenging hedgehog carcases is highly plausible. *Polymorphus* species, on the other hand, are only known to exhibit an aquatic life cycle, thus making infection of a hedgehog less probable. The finding of Smales et al. (2010) was the only occurrence on record, so this is a presumably a rare phenomenon. The *Polymorphus* sp. specimen in question possessed 20 rows of 5–6 hooks, thus was not the same species as *P. circi* **n. sp.**

The terrestrial lifestyle of the harrier raises the question of a potential intermediate host for *Polymorphus circi* **n. sp.** The intermediate host of *Polymorphus* species is always a crustacean; most usually an amphipod or occasionally a crayfish (Crompton and Nickol 1985). If the life cycle of this new species is completed in freshwater, the only crustacean large enough to count as prey would be the native crayfish, *Paranephrops zealandicus* (White), found on Stewart Island and on the eastern side of South Island (Whitmore et al. 2000). Freshwater crayfish are not unknown as intermediate hosts for *Polymorphus* species: *P. biziurae* Johnston & Edmonds, 1948 (parasite of the musk duck *Biziura lobata* in Australia) and *P. boschadis* (Schrank, 1788) [now *P. minutus*] (parasite of Anatidae in Europe) have been found in *Cherax destructor* (Clark) (Edgerton et al. 2002). If the life cycle of this species is completed in marine water, the intermediate host would likely be an amphipod. Although there are records of harriers feeding on crabs in intertidal areas (Latham 2002; Totterman 1997) they are unlikely to be intermediate hosts for this acanthocephalan. Nickol et al. (1999) reviewed the morphology of all species assigned to *Polymorphus* that were found as cystacanths in crabs and concluded “it appears that all known records of *Polymorphus* in decapods refer to species of the subgenus *Profilicollis*...” (Nickol et al. 1999), thereby ruling out crabs as potential hosts of *Polymorphus*. The most likely contender for intermediate host seems to be terrestrial amphipods (Talitridae). There are 28 species of talitrid in New Zealand; they are found in damp habitats and make up a significant component of the leaf litter and soil fauna (Fenwick & Webber 2008). Unfortunately, the origin of our infected birds does nothing to clarify the question of intermediate host: of the three birds from a known locality, two were found on the Taeri Plain (an inland low-lying flood plain with rivers, lakes and marshland) and one was found almost adjacent to the marine water at the port of Timaru. Further examination of likely arthropod contenders from relevant areas is ongoing.

The literature review required for this study highlighted some discrepancies in current species descriptions. Four species from Pakistan are *incertae sedis* as no trunk spines are recorded in their descriptions (*P. fatimaae* Khan, Dharejo, Birmani and Bilqees, 2008*, P. mohiuddini* Muti-ur-Rahman, Khan, Bilqees and Khatoon, 2008*, P. nickoli* Khan and Bilqees, 1998 and *P. sindhensis* Khan, Ghazi and Bilqees, 2002). We have not seen examples of these species so refrain from reallocating them, but a number of features in the descriptions suggest them to be species of *Centrorhynchus*. These specimens require re-examination to place them in the correct genus.

There are many reports in the literature of acanthocephalans causing pathological symptoms or death to their hosts. The proboscis burrows into the intestinal wall where it is secured by the proboscis hooks, in order to hold the parasite in its preferred site in the gut. The lesions thus caused may affect innervation of the intestine, as well as secretory and motor function (Petrochenko 1958). In some cases, the whole worm may burrow right through the intestine and come to rest in the body cavity resulting in potentially lethal peritonitis (Clark et al. 1958). In very severe infections, or when birds are otherwise health-compromised, *Polymorphus* spp. have been implicated in mass fatalities among bird hosts (Crompton and Nickol 1985). Although none of the infections found in harriers was sufficient to have been the cause of death, in the light of the potential for pathogenicity provided by these acanthocephalans it is advised that conservation and animal health practitioners in New Zealand are alert to the occurrence of *Polymorphus circi* **n. sp.** in harriers or other potential hosts.

## Data Availability

Not applicable.
